# Macroscopic and Mesoscopic Deterioration Behaviors of Concrete under the Coupling Effect of Chlorine Salt Erosion and Freezing–Thawing Cycle

**DOI:** 10.3390/ma14216471

**Published:** 2021-10-28

**Authors:** Shaojie Chen, Jianxi Ren, Yugen Li, Xiang Ren, Yongjun Song, Jielong Sun

**Affiliations:** 1 School of Architecture and Civil Engineering, Xi’an University of Science and Technology, Xi’an 710054, China; renjianxi1968@163.com (J.R.); renxiang798@xust.edu.cn (X.R.); songyj79@xust.edu.cn (Y.S.); 2 School of Architecture Engineering, Yulin University, Yulin 719000, China; liyugen@yulinu.edu.cn; 3 School of Architecture Engineering, Yan’an University, Yan’an 716000, China; sunjielong@126.com

**Keywords:** concrete, chlorine salt erosion, freezing–thawing cycle, microstructure, damage

## Abstract

The aim of this study was to reveal the macroscopic and mesoscopic deterioration behaviors of concrete under the coupling effect of chlorine salt erosion and the freezing–thawing cycle. The rapid freezing–thawing test was carried out in a 5% chlorine salt environment. The macroscopic characteristics of concrete were analyzed by testing the mass, the relative dynamic modulus of elasticity, and the compressive strength of concrete under different freezing–thawing cycles. Using CT scanning technology and three-dimensional reconstruction technology, the pore structure, CT value, and surface deviation of concrete before and after freezing–thawing were analyzed. Based on the changes of solid volume, pore volume, and solid CT value of concrete, the calculation method of relative CT value was proposed, and the damage model was established with relative CT value as the damage variable. The results demonstrate that the mass loss rate decreases in the beginning and then increases in the process of chlorine salt erosion and freezing–thawing, and the smaller the concrete size, the greater the mass loss rate. The relative dynamic modulus of elasticity decreases gradually, slowly at the initial stage and then at a faster rate, and the compressive strength loss rate increases gradually. The pore quantity, porosity, and volume loss rate of concrete increase in a fluctuating manner, whereas the relative CT value decreases. The comprehensive analysis shows that the chlorine salt frost resistance of concrete is negatively related to the water-cement ratio when the freezing–thawing cycle is fixed. The damage model could better reflect the freezing–thawing damage degree of concrete with different water cement ratios, and the damage evolution process is well described by the Weibull function.

## 1. Introduction

With the advantages of simple production technology, easy availability of raw materials, wide range of strength grade, and low cost, concrete has become one of the most extensively used building materials [1]. It has been widely used in buildings, hydraulic engineering, bridges, roads, and other engineering applications [2]. However, increasing problems in the durability of concrete structures have been reported due to the complicated service environments [3]. For example, in cold regions, the positive and negative alteration of temperature is one of the important causes of durability deterioration of concretes. Moreover, such freezing–thawing cycles usually do not exist independently [4]. Additionally, buildings in salinized soil regions, concrete structures in coastal regions, and concrete road and bridge structures that use deicing salts often bear the compounded effect of salt erosion and freezing–thawing cycles, resulting in various durability problems, including peeling of concrete cover, rebar exposure, and rebar erosion [5].

Chlorine salt erosion and freezing–thawing damage are important factors that influence concrete durability, thereby significantly affecting structural performance and service life [6]. In chlorine salt environments, Cl^−^ spreads continuously in concrete. On the one hand, Cl^−^ induces chemical reactions with hydration products in concrete, loosening the internal structures of concrete materials [7]. On the other hand, Cl^−^ induces erosion of rebar, thus causing structural damages [8]. Influenced by positive–negative alterative changes of temperature, concrete porosity changes and the permeability is influenced, thus changing the concrete’s mechanical properties [9]. Under the coupling effect of salt erosion and freezing–thawing cycles, the damage mechanism of concrete becomes more complicated. Traditional studies have mainly focused on the deterioration behaviors of concrete under a single environmental factor. Nevertheless, the engineering field environment is often influenced by both salt erosion and freezing–thawing cycles. Different factors might exert a collaborative effect on concrete deterioration, thus intensifying the damages [10,11]. Therefore, it is necessary to study the deterioration laws of concrete durability under the coupling effect between chlorine salt erosion and freezing–thawing cycles.

Currently, a great deal of research on concrete durability under the influences of salt erosion and/or freezing–thawing cycles has been done. Under chlorine salt erosion, Li et al. [12] characterized the porosity variation characteristics of concrete after chlorine salt erosion by the scanning electron microscope (SEM) and discussed the relationship between the microscopic and macroscopic performances of concrete after such an erosion. Koga et al. [13] carried out an experimental study on salt erosion resistance of Belite-Ye’elimite-Ferrite (BYF) cement-based materials, in which diffusion laws of Cl^−^ were analyzed, and the durability was evaluated. Referring to the variation laws of concrete mass and compressive strength, Mangi et al. [14] discussed the influences of fly ash on concrete durability in the chlorine salt and sulfate environment. With respect to the freezing–thawing cycle, Gong et al. [15] established a hydraulic model by studying the water permeability laws of concrete under several freezing–thawing cycles and evaluated the freezing damage degree using the permeability coefficient. Qiu et al. [16] analyzed freeze resistance of coal gangue concrete from mass, compressive strength, and elasticity modulus and established a damage evolution equation based on acoustic emission features in the process of concrete compression failure. Some scholars have studied the effect of activated pozzolan on hydration and microstructure of natural pozzolan paste [17]. Liu et al. [18] have discussed the effects of nanoparticles on the durability and internal degradation of concrete. The effects of different particle sizes, different nanoparticle contents, and different nanoparticle types on the freezing–thawing resistance, pore size, distribution, and degradation performance of concrete have also been analyzed. The results show that the particle size and dosage of nanoparticles have a great influence on the pore distribution of concrete in the freezing–thawing cycle. However, these studies mainly focused on the influencing laws of salt erosion or freezing–thawing cycles on concrete durability.

Some scholars have studied concrete durability under the coupling effect of chlorine salt erosion and freezing–thawing cycles. For example, An et al. [19] carried out a salt freezing–thawing cycle test of concrete with 0.2 water–cement ratio and analyzed the variation laws of bound water and porous structure before and after the freezing–thawing cycle. They also discussed the damage mechanism of concrete by combining the ice expansion rate of an NaCl solution. Skripkiūnas et al. [20] carried out a durability test of eight concrete samples with different water–cement ratios in 3% NaCl solution and discussed the influences of cement on concrete’s salt freezing resistance. Based on the mechanical theory of fatigue damages, Yu et al. [21] deduced a damage model of concrete under salt freezing conditions by using the loss rate of dynamic modulus of elasticity as the damage variable. However, the aforementioned studies evaluated concrete durability from the macroscopic aspects, such as mass, compressive strength, and relative dynamic modulus of elasticity (RDME) [22,23,24,25]. In fact, it is difficult to thoroughly disclose the deterioration mechanism of concrete under salt freezing-induced damages through the macroscopic perspective alone. Therefore, some scholars have analyzed the evolutionary process of concrete’s internal structures from a microscopic perspective. For instance, Henry et al. [26] carried out a nondestructive test on the internal microstructure of high-strength concrete by computed tomography (CT) technology and analyzed the influences of heating and re-curing on microstructural characteristics. Suzuki et al. [27] analyzed the fracture distributions of concrete after the freezing–thawing cycle by CT technology and evaluated concrete damage by combining acoustic emission features.

Hence, CT technology could characterize the evolutionary characteristics of concrete’s internal materials and pores after being influenced by the external environment in a more straightforward and precise manner. This study analyzed the relationship between microscopic characteristics and macroscopic indexes with CT technology as a mesoscopic characterization method, combined with the macroscopic deterioration law of concrete under the chloride salt erosion and freezing-thawing. Based on the changes of volume, pore structure, and CT value, the relative CT value was used to characterize the damage of concrete, and the degradation mechanism of concrete with different water cement ratios under the chlorine salt erosion and freezing–thawing cycle was comprehensively analyzed.

## 2. Test Materials and Methodology

### 2.1. Test Materials

In the test, the ordinary Portland cement P·O 42.5 from Jidong Cement (Tongchuan Co., Ltd., Guangzhou, China) was used. The physical and mechanical properties are listed in Table 1. The coarse aggregate was detrital limestone with the diameter range of 5–20 mm, an apparent density of 2750 kg/m^3^, and a crushing index of 6%. The fine aggregate was medium sand, with an apparent density of 2630 kg/m^3^, a mud content of 1.1%, and a fineness modulus of 2.7. The material parameters of gravel and sand were obtained according to the test method of Chinese Standard JGJ 52 [28]. Local tap water was used for mixing the concrete.

### 2.2. Mixing Ratio

Concrete specimens with three different water–cement ratios were designed in the test. All specimens were divided into groups A, B, or C according to different water–cement ratios, and each group included two sizes of specimens. There were three 100 mm × 100 mm × 400 mm specimens in each group, and the samples were used to test the dynamic modulus of elasticity. Thirty-nine 100 mm × 100 mm × 100 mm specimens in groups A and B and twenty-seven 100 mm × 100 mm × 100 mm specimens in group C were used to test the compressive strength of concrete, and three specimens in each group were taken out every 25 freezing–thawing cycles. Mixing ratios and mechanical properties are listed in Table 2. The concrete was cured for 24 h in mold and then demolded. The specimens were then moved to a curing box under standard curing conditions with a humidity of 95% and a temperature of (20 ± 2) °C based on the Chinese Standard GB/T 50081 [29] for 28 days.

### 2.3. Methodology

The freezing–thawing test was conducted using the fast-freezing method in *Standard for test methods of long-term performance and durability of ordinary concrete* (GB/T 50082-2009) [30]. In the freezing–thawing cycle test, the KDR-V9 concrete fast freezing–thawing cycle machine (manufacturer by Digital Intelligence Yilong Instrument Co., LTD., Beijing, China) was used. The cycle system consisted of one freezing–thawing cycle, which was finished in 2–4 h. The freezing and thawing temperatures were controlled at −18 ± 2 °C and 5 ± 2 °C, respectively, and the thawing time was no shorter than 1/4 of the cycle.

The concentration of salt solution has an influence on the frost resistance of concrete. Yang [31] showed that the frost resistance of concrete was the most unfavorable when the concentration of the NaCl solution was 2–6%. Current research on the erosion of NaCl solution generally selects 3.5% [32] and 5% [33]. This study mainly focused on the macroscopic and mesoscopic damage degradation law of concrete with different water cement ratios under chloride salt and freezing–thawing cycles, and we selected a 5% NaCl solution as the medium.

**Figure 1:**
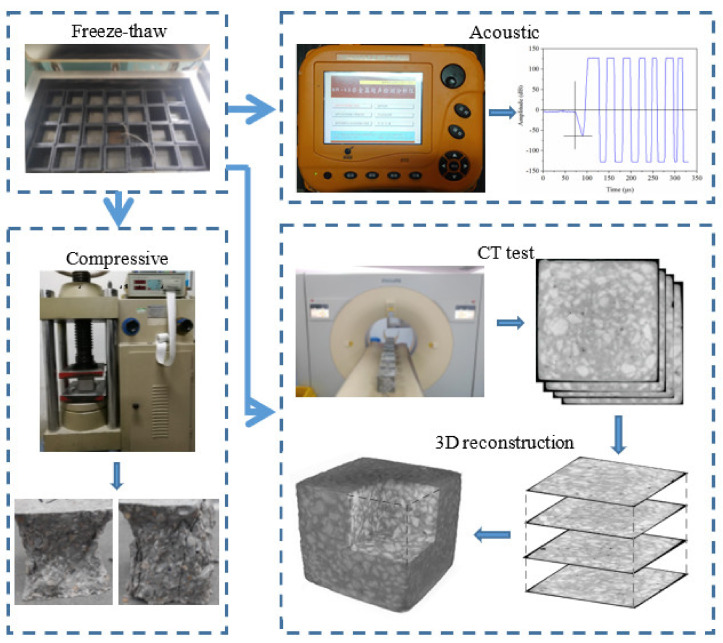
Major test and analysis process.

First, three groups of specimens were immersed in 5% (mass fraction) NaCl solution for 4 d, and the freezing–thawing cycle test was performed in 5% NaCl solution after attaining saturation. The NM-4B nonmetallic acoustic wave tester (manufacturer by KONCRE TE Engineering Testing Technology Co., LTD., Beijing, China) was used to test the wave speed of concrete prismoid, after which the RDME was calculated. The Brilliance iCT 256 hierarchical spiral topspeed CT machine (manufacturer by Philips, Netherlands) was used for chip scanning of cube specimens. Scanning parameters were set as follows: slice thickness: 1 mm, display matrix: 512 × 512, voltage: 120 kV. CT tests were conducted under saturation conditions of concrete. To avoid liquid evaporation, specimens were wrapped with plastic film. After obtaining CT images, the specimens were rebuilt by VGStudio Max 3.2 (Volume Graphics GmbH, Heidelberg, Germany). After setting unified coordinates, the pore structures, CT values, and other mesoscopic indicators were obtained through threshold segmentation method. The major test process is shown in Figure 1.

## 3. Test Results and Analysis

### 3.1. Evolutionary Rules of the Macroscopic Characteristic of Concrete

#### 3.1.1. Morphology

**Figure 2:**
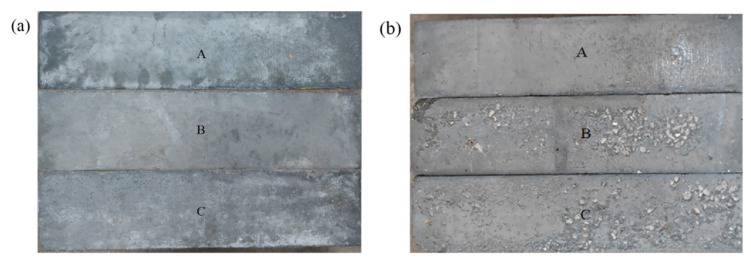

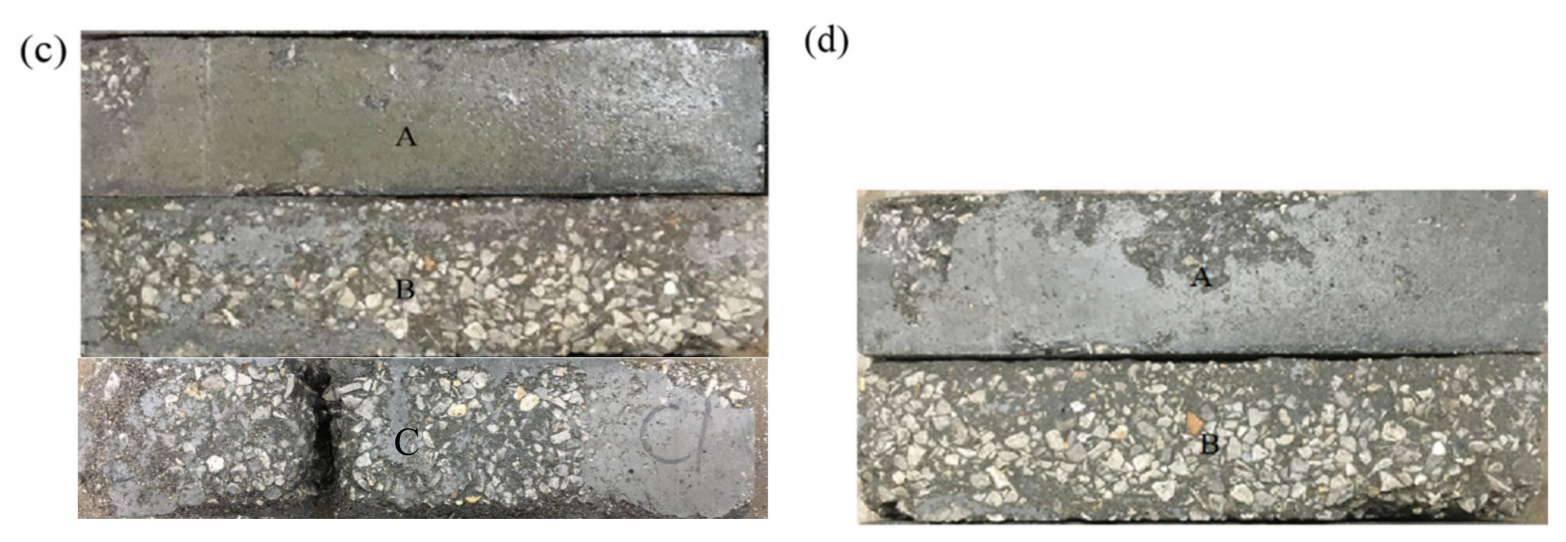
Concrete morphology: (**a**) 0; (**b**) 100; (**c**) 200; and (**d**) 300 salt freezing–thawing cycles.

Morphologies of concretes with different water–cement ratios after different salt freezing–thawing cycles are shown in Figure 2.

As can be seen, after 100 salt freezing–thawing cycles, Group A had small surface damage, whereas Groups B and C began to develop mortar peeling on the concrete surface. After 200 salt freezing–thawing cycles, the surface damage of concrete became intensified. Group A showed evident surface mortar peeling at the corners. Group B showed serious surface mortar peeling on the surface and exposed aggregates. Group C showed the most serious damages and a large aggregate exposure range accompanied by aggregate falling at the corners. Moreover, one specimen from Group C developed rupture failure. After 300 salt freezing–thawing cycles, the surface mortar peeling range of Group A was expanded, and aggregates at the corners fell. The surface mortar peeling of Group B was intensified accompanied by evident aggregate falling at the edges and corners. Furthermore, Group B could not maintain the integral morphology. Obviously, the apparent damage of concrete, which was greatly influenced by the water–cement ratio, was intensified gradually with the increase in salt freezing–thawing cycles. The higher water–cement ratio led to the large and rapid development of apparent damage. The salt freezing-induced damage of concrete mainly presented the following phenomenon: surface mortar peeling in the early stage of salt freezing, progressive inward damage and aggregate falling with increased salt freezing–thawing cycles, and finally rupture failure.

#### 3.1.2. Mass Loss Rate

**Figure 3:**
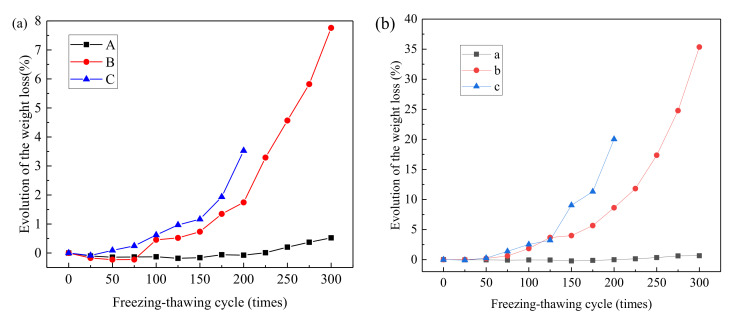
Mass loss rates of concretes: (**a**) prismoid specimens; (**b**) cube specimens.

The mass variation law of concrete can be reflected by solution absorption and peeling degree during salt freezing–thawing cycles. The mass loss rates of different groups of prismoid samples are shown in Figure 3a. As can be seen, the mass loss rate of all groups decreased in the early period of salt freezing, indicating that the concrete’s solution absorption mass was higher than surface peeling mass at this stage. This can be attributed to relatively low concrete damage and small surface peeling mass in the early period. However, the concrete structure was loosened, and solution absorption mass increased due to the freezing–thawing cycles, thus bringing a negative growth of mass loss rate. After 100 salt freezing–thawing cycles, the mass loss rates of prismoid specimens in Groups A, B, and C were −0.13, 0.45, and 0.62%, respectively. The solution absorption mass of Group A was higher than the surface peeling mass. However, the mass loss rates of Groups B and C were both positive, indicating that the surface peeling mass of concrete caused by salt freezing was higher than the solution absorption mass. After 200 salt freezing–thawing cycles, the mass loss rates of prismoid specimens in Groups A, B, and C were −0.08, 1.74, and 3.53%, respectively. Obviously, the mass loss rate of all groups increased to some extent, but the solution absorption mass of Group A was still higher than the surface peeling mass. After 300 salt freezing–thawing cycles, the mass loss rates of Groups A and B were 0.52 and 7.76%, respectively. Hence, the mass loss rate continued to increase.

The mass loss rates of cube specimens are shown in Figure 3b. As can be seen, the variation laws of the mass loss rate of cube specimens were consistent with those of prismoid specimens. However, given the same salt freezing–thawing cycles, great differences in mass loss rates were observed between cube and prismoid specimens. After 200 salt freezing–thawing cycles, the mass loss rates of Groups A, B, and C were −0.02, 8.63, and 20.03%, which were about 0.25, 4.96, and 5.67 times those of prismoid specimens, respectively. After 300 salt freezing–thawing cycles, the mass loss rates of Groups A and B were 0.65 and 35.36%, which were 1.25 and 4.56 times those of prismoid specimens, respectively. According to the analysis, the mass loss rate of concrete decreased first and then increased with the increase in salt freezing–thawing cycles. In addition, the mass loss rate increased further, and the growth rate was higher when the water–cement ratio increased. Moreover, there was an evident size effect of mass loss rate of concrete in the salt freezing–thawing cycles, in which the mass loss rate was higher if the size was smaller.

#### 3.1.3. RDME

**Figure 4:**
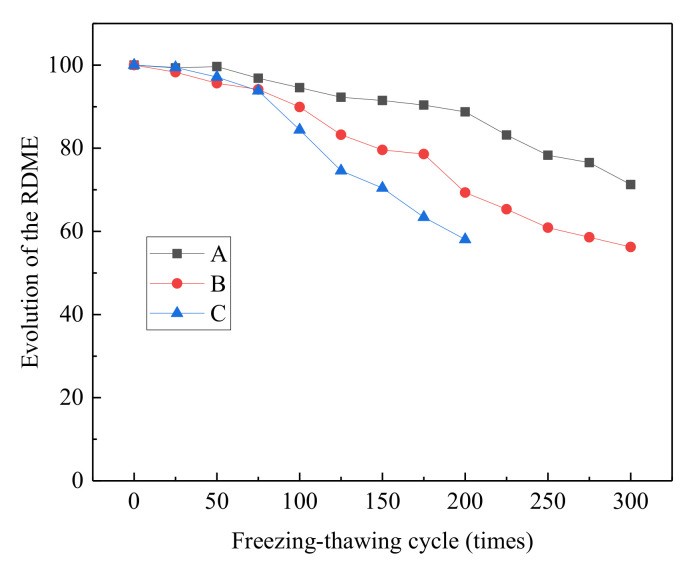
RDME of concrete.

The different curves of the RDME of different concrete groups under salt freezing–thawing cycles are shown in Figure 4. A small difference among different groups was initially observed in terms of RDME before 75 salt freezing–thawing cycles. After 100 cycles, the three groups showed great differences in RDME. With the increase in salt freezing–thawing cycles, the reduction rate of RDME increased. Group C showed the highest reduction amplitude, followed by Groups B and A successively. After 100 cycles, RDME of Groups A, B, and C decreased to 94.56, 89.92, and 84.43%, respectively. After 200 cycles, the RDME of Groups A, B, and C decreased to 88.74, 69.34, and 58.07%, respectively. After 300 cycles, the RDME of Groups A and B decreased to 71.27 and 56.25%, respectively. According to *Long-term Performance and Durability Test Method of Ordinary Concrete* (GB/T 50082-2009), the judgment reference of failure occurs when the RDME decreases to 60%. In the current study, Group C first broke at 200 salt freezing–thawing cycles and Group B broke at 250 cycles.

As shown in Figure 4, the RDME of concrete decreased first with the increase in the number of salt freezing–thawing cycles. The reduction amplitude was initially slow and then became faster. Moreover, the reduction amplitude and reduction rate of RDME were positively related to the water–cement ratio. This variation law of RDME can be interpreted as follows. In the early period of salt freezing–thawing cycles, concrete developed some damage, but most of them were on the surface with only small damage inside. As a result, the RDME changed slightly in the early stage. With the increase in the number of salt freezing–thawing cycles, the damage progressed gradually from the surface to the inside part of the concrete, thus increasing variations of RDME.

#### 3.1.4. Compressive Strength Loss Rate

**Figure 5:**
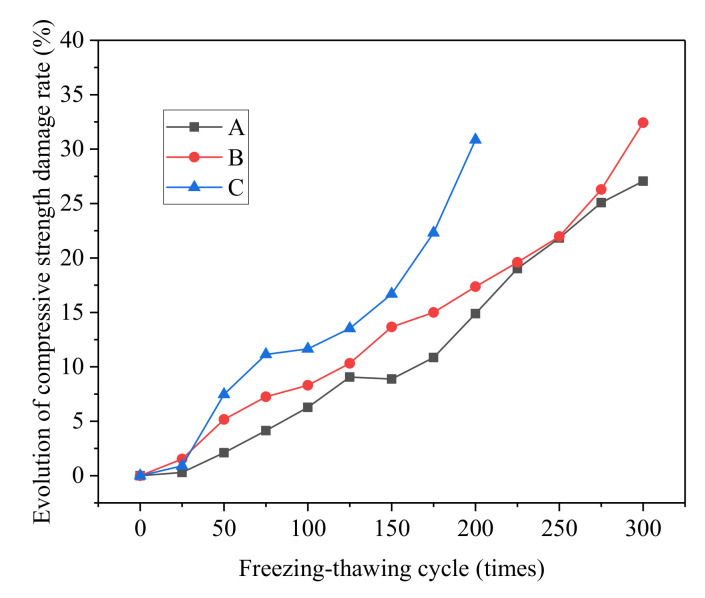
Compressive strength loss rate of concretes.

Variations of compressive strength loss rate of different groups under salt freezing–thawing cycles are shown in Figure 5. As can be seen, after 100 salt freezing–thawing cycles, the compressive strength loss rates of Groups A, B, and C were 6.27, 8.30, and 11.65%, whereas after 200 salt freezing–thawing cycles, the loss rates of Groups A, B, and C were 14.88, 17.36, and 30.86%, respectively. After 300 cycles, the loss rates of Groups A and B were 27.06 and 32.43%, respectively. According to the test results, the overall compressive strength loss rate of all groups increased gradually with the increase in salt freezing–thawing cycles. Moreover, compressive strength loss rate was positively related to the water–cement ratio.

The reduction of concrete strength was introduced as follows. Chlorine crystal pressure and the expansive force of solution acted on the micropores in the concretes. When the pore wall pressure exceeded the compressive strength of concrete, cracks initiated inside the concrete [34]. With the increase and propagation of cracks, connecting cracks were formed, gradually weakening regions of the concrete. These weak regions finally formed a failure surface at the compressive failure of concrete, thus decreasing the material’s strength. Given that the internal structure of concrete with smaller water–cement ratios was more compact, the tensile strength of the materials was high. Hence, the concrete developed fewer cracks later in the salt freezing–thawing cycles, and showed less damage. Given the same salt freezing–thawing cycles, compressive strength loss rate was lower than that of concrete with a high water–cement ratio.

### 3.2. Evolution Rules of Concrete Microstructure

#### 3.2.1. CT Image Analysis

**Figure 6:**
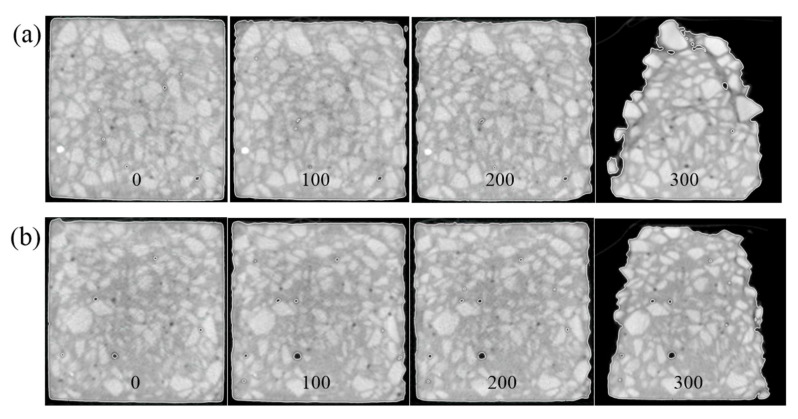

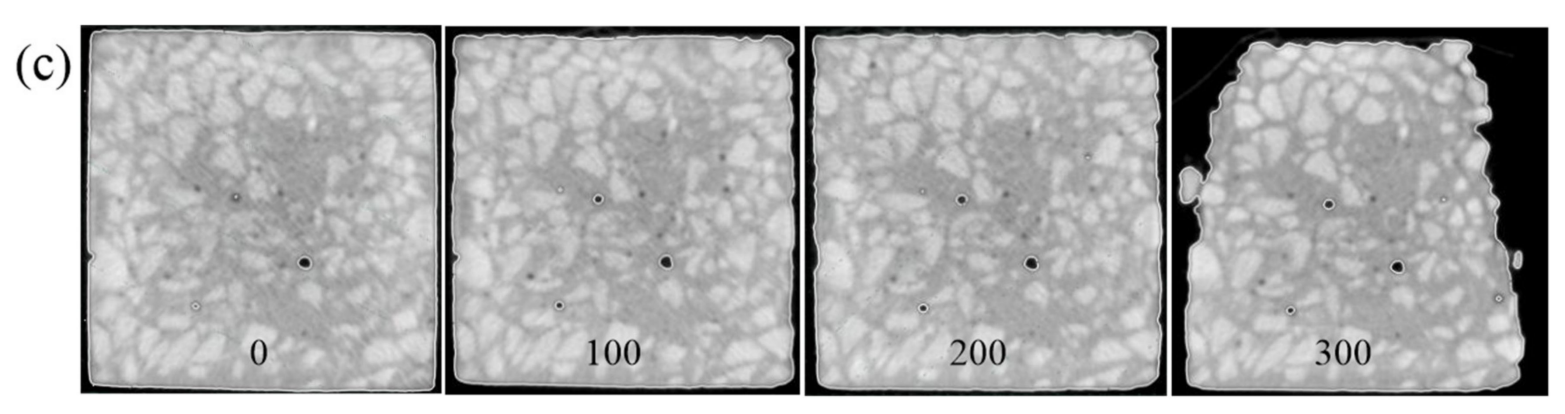
CT section images of concrete: (**a**) section 1; (**b**) section 2; (**c**) section 3.

To study the evolution rules of the internal microstructure of concrete with different water–cement ratios under salt freezing–thawing cycles, the CT section images of concrete after salt freezing–thawing cycles were analyzed in this study. Limited by the article length, only Group B was analyzed. Three sections were chosen for display (Figure 6). Aggregate, mortar, and pore distributions in concrete sections can be seen intuitively from Figure 6. Under salt freezing–thawing cycles, pores in the core zones of the concrete sections changed slightly, but some pores at the external regions of the section were narrowed and even disappeared. This is mainly because chlorine salts that penetrated the concrete gradually expanded during the salt freezing–thawing cycles, thus filling in some pores. The loss of mortar and aggregates in concrete sections evolved from the surface to inside. With the increase in salt freezing–thawing cycles, the material falling at the external sides of concrete sections increased gradually. When the number of salt freezing–thawing cycles increased to 300, there occurred serious external damage on the concretes, and new cracks initiated along the pores and interface zones at the external sides of sections. These internal microcracks propagated and connected gradually to cause aggregate falling along the cracks. With the increase in the number of salt freezing–thawing cycles, the damages extended inward gradually. Eventually, concrete aggregates fell off from outside to inside, and the structures were acidized.

**Figure 7:**
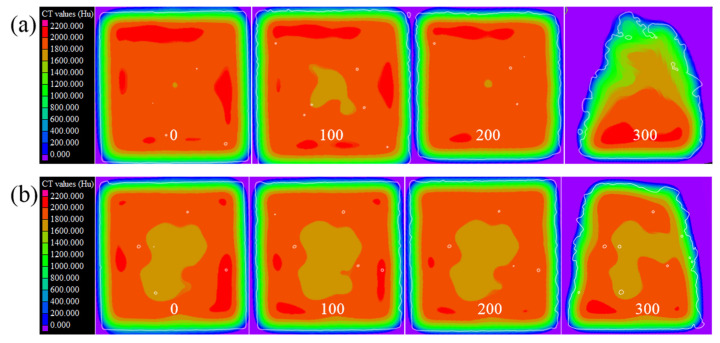

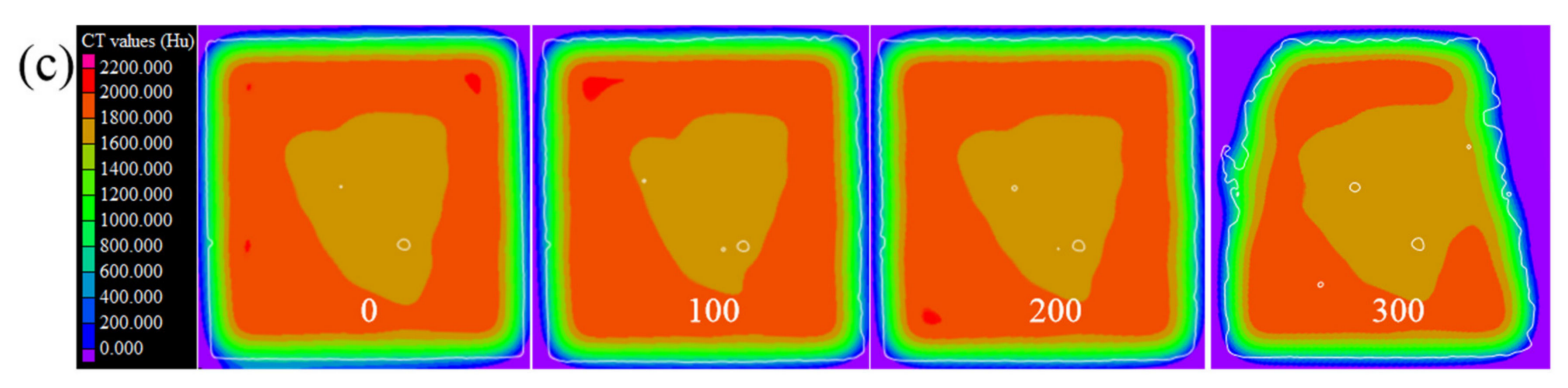
CT value hotspot distributions on concrete sections under salt freezing–thawing cycles: (**a**) section 1; (**b**) section 2; (**c**) section 3.

Evolution of internal structures could be analyzed by observing the sections. However, it is difficult to judge the variation laws of compaction degree in different regions of concrete. To observe the evolution of the compaction of concrete section more intuitively, the distribution diagrams of CT value hotspots on different sections are shown in Figure 7. CT value at the external sides of the concrete sections was higher than that in other regions. This may be due to the aggregates being relatively concentrated on the external side of the concretes and the higher compaction compared to that in other regions. After the salt freezing–thawing cycles, CT values on the external sides of the concrete increased first and then decreased, but they generally decreased continuously in the internal core zone. The different variation laws of CT values in the internal and external regions of the concrete samples proved the macroscopic failure features. In the early stage of the salt freezing–thawing cycles, pores in the external regions of concrete became more compacted when the crystalline salts filled the pores, thus increasing the CT value. In the late stage, the concrete damages were intensified, and the structures were loosened gradually due to the collaborative effect of crystal pressure and frost heaving pressure, thus decreasing CT value. With the continuous increase in salt freezing–thawing cycles, the external aggregates began to fall gradually, and the damages progressed inward. As the concrete compaction degree in the core zones decreased gradually, the CT value inside also decreased gradually.

#### 3.2.2. Three-Dimensional Reconstruction

**Figure 8:**
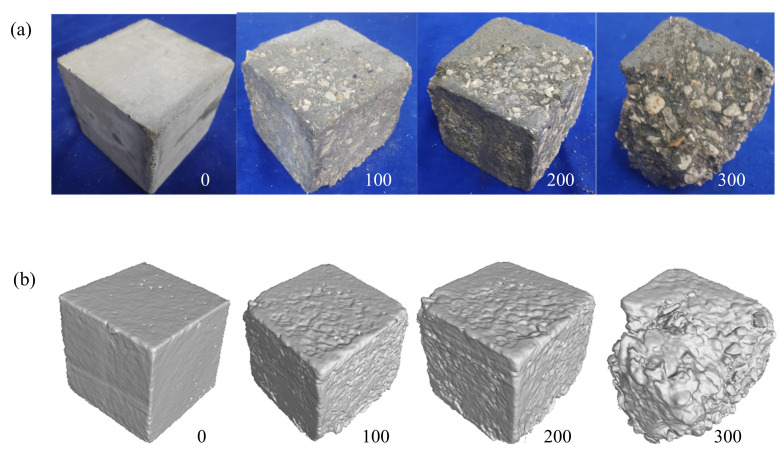
Morphologies and 3D reconstruction model of concrete: (**a**) morphologies; (**b**) 3D reconstruction model.

In this study, three-dimensional (3D) reconstruction of CT scanning data was conducted by using VGStudio Max software. The microstructural evolution rules of concrete after the salt freezing–thawing cycles were analyzed through variations in meso-structure parameters, such as pore structure, rebuilt volume, CT value, and surface deviation. Let us take Group B as an example. The morphologies and 3D reconstruction model after 0, 100, 200, and 300 salt freezing–thawing cycles are shown in Figure 8.

As shown in Figure 8a, cube concretes experienced a gradual damage progress, including mortar peeling, aggregate exposure, and aggregate falling at the corners under salt freezing–thawing cycles. The 3D reconstruction model in Figure 8b accurately reflects the specimen morphologies after different salt freezing–thawing cycles. This provides guarantee for the subsequent analysis of parameters.

After 3D reconstruction, the quantity of pores, porosity, volume, and CT value of concrete could be ascertained, thus facilitating the analysis of the evolutionary rule of concrete microstructure. The selection method is mainly determined by the practical situations of the chosen concrete, and the reasonable threshold range is acquired through repeated debugging. The chosen pore threshold range in the current study was –1024 to 1250 Hu, and the threshold range of solid phase materials was 1250 to 3701 Hu.

#### 3.2.3. Pore Structures

**Figure 9:**
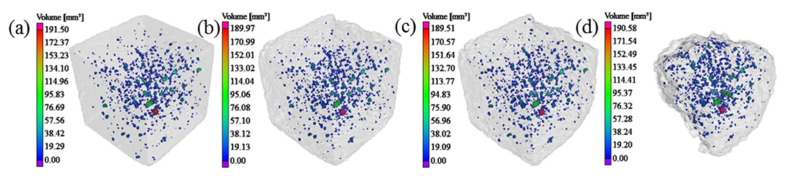
Pore structures in concrete: (**a**) 0; (**b**) 100; (**c**) 200; and (**d**) 300 salt freezing–thawing cycles.

The pore structures of concrete according to the chosen threshold ranges are shown in Figure 9. The gray profile represents the overall morphology of concrete. Regions in different colors in the profile represent pores of different volumes. The left tag reflects volume size of different colors. Clearly, pores at the external side of concrete disappeared with the material falling under salt freezing–thawing cycles. However, the number of pores increased in the external region of concrete after material falling and some pores were connected.

**Figure 10:**
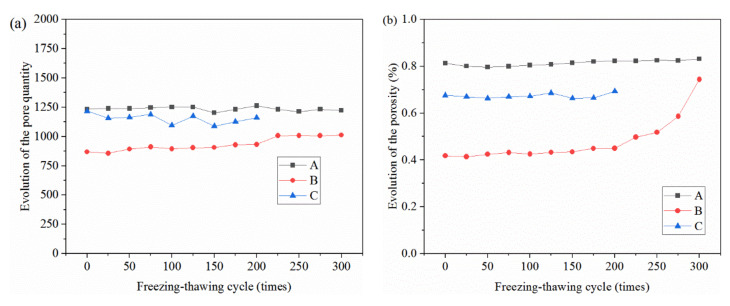
Evolutionary rules of pore quantity and porosity of concrete: (**a**) pore quantity; (**b**) porosity.

According to the pore extraction results, the quantity and porosity of pores of concrete after the salt freezing–thawing cycles was analyzed. The evolutionary rule of the pore quantities and porosity of different concrete groups is shown in Figure 10.

As shown in Figure 10a, there were some differences in pore quantity among the different groups. With the increase in salt freezing–thawing cycles, pore quantity also increased in a fluctuating manner. In the whole process of salt freezing–thawing cycles, the increasing amplitudes of the pore quantities of Groups A and B were relatively small, and the increasing amplitudes of pore quantities of Group C in the late stage increased significantly. As concrete aggregates were loosened, and new cracks were initiated in a high quantity, this resulted in fluctuations in the salt freezing–thawing cycles. When salt freezing-induced damages developed to a certain degree, small pores were connected gradually into large ones and even induced material falling. As a result, pores in the falling region disappeared and pore quantity decreased. During the salt freezing–thawing cycles, pore initiation, connection, and disappearance in concrete occurred repeatedly, thus resulting in the fluctuation law. However, pore quantity increased continuously with the intensified degree of damage.

The evolutionary rules of the concrete porosities of different groups based on the number of salt freezing–thawing cycles were analyzed (Figure 10b). It can be seen from Figure 10b that after 100 salt freezing–thawing cycles, the porosity of Group A reduced by 0.008%, and the reducing amplitude was 0.94% compared with initial porosity. The porosity of Group B increased by 0.007%, and the increasing amplitude was 1.76%. The porosity of Group C increased by 0.004%, and the increasing amplitude was 0.65%. After 200 salt freezing–thawing cycles, the porosities of Groups A, B, and C increased by 0.006%, 0.032%, and 0.017%, respectively, and their corresponding increasing amplitudes were 0.64, 7.72, and 2.51%. After 300 salt freezing–thawing cycles, the porosities of Groups A and B increased by 0.019 and 0.327%, respectively, and their corresponding increasing amplitudes were 2.30 and 78.26%. According to the test results, the overall porosity of all groups increased gradually throughout the salt freezing–thawing cycles, increasing slowly at first and then quickly afterwards. In the late stage of salt freezing–thawing cycles, the porosity kept increasing, although some pore volume was lost due to concrete falling in some regions.

#### 3.2.4. Volume Loss Rate

**Figure 11:**
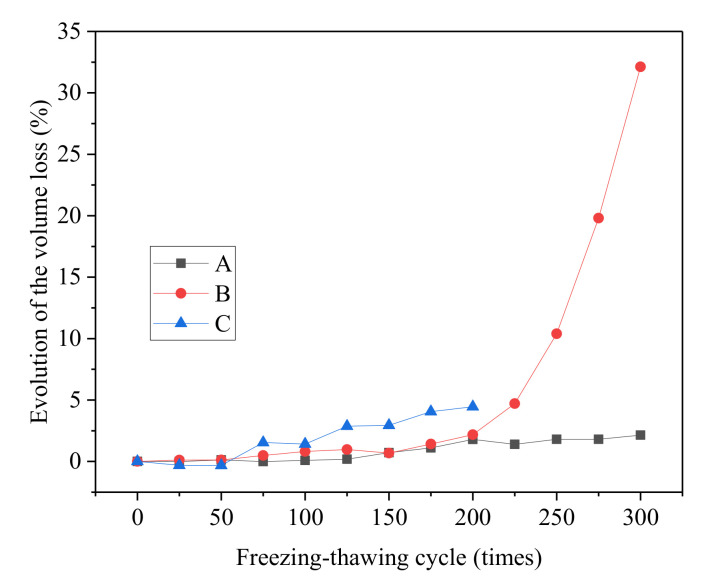
Evolutionary rules of volume loss rates of different groups.

After 3D reconstruction, the volume information of scanned concrete can be gained. Based on the above morphological analysis results, concrete shows great material loss after the salt freezing–thawing cycles. The volume loss rates of the concrete specimens were analyzed to conduct a quantitative study on concrete volume loss of different groups under salt freezing–thawing cycles (Figure 11).

With the increase in the number of salt freezing–thawing cycles, the volume loss rate of different groups changed greatly. After 100 cycles, the volume loss rates of Groups A, B, and C were 0.09, 0.82, and 1.42%, respectively. After 200 cycles, the volume loss rates of Groups A, B, and C were 1.80, 2.18, and 4.46%, respectively. After 300 cycles, the volume loss rates of Groups A and B were 2.14 and 32.12%, respectively. In summary, the volume loss rate of concrete increased gradually with the increase in the number of salt freezing–thawing cycles. Given the same salt freezing–thawing cycles, overall volume loss rate of concrete was positively related with the water–cement ratio.

#### 3.2.5. CT Value

**Figure 12:**
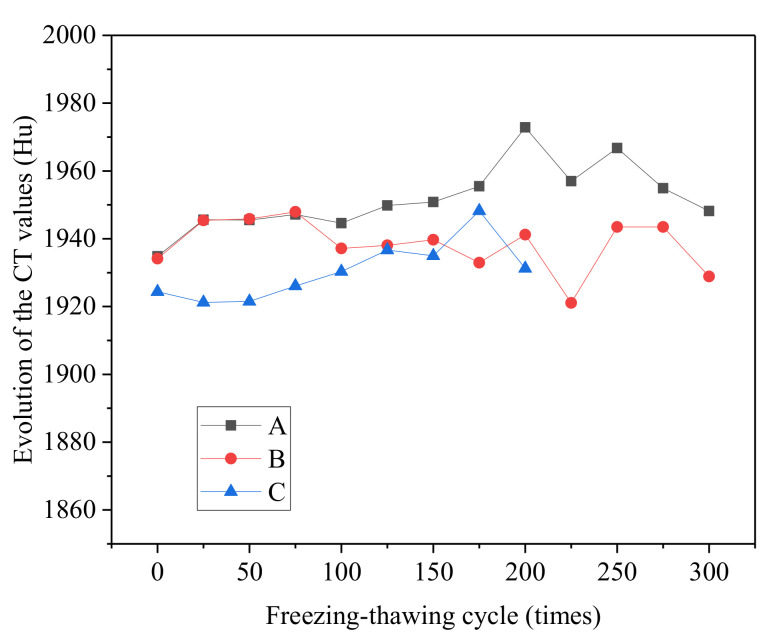
Evolutionary rules of the CT values of solid phases of concrete in different groups.

As shown in the results, concrete experienced changes in compaction degree in addition to changes in pore structures and volume under salt freezing–thawing cycles. Among all parameters measured from the CT test, CT value is the parameter that reflects the compaction degree of materials. Therefore, the variation laws of the compaction degrees of concrete specimens were analyzed by CT values. The evolutionary rules of solid CT values of concrete are shown in Figure 12.

As shown in Figure 12, there was no monotonous increasing and decreasing relations between the solid CT value of concrete and the number of salt freezing–thawing cycles. The fluctuation of solid CT can be mainly attributed to different CT values of components of solid materials. Furthermore, the CT value of mortar in concrete was lower than that of coarse aggregates. When mortar peeled off, the proportion of aggregate volume in the remaining parts of the materials increased, thus increasing the solid CT value. When aggregates peeled off, the proportion of mortar volume in the remaining parts of the materials increased, thus decreasing the solid CT value. Solid CT value can reflect the compaction degree of the remaining parts of the materials after a salt freezing–thawing cycle, and the variation law of solid CT value could reflect the lost material components of concrete. It is necessary to conduct a comprehensive analysis by combining this with other parameters in order to analyze damage evolutionary rules of concrete in the salt freezing–thawing cycles.

#### 3.2.6. Surface Damages

**Figure 13:**
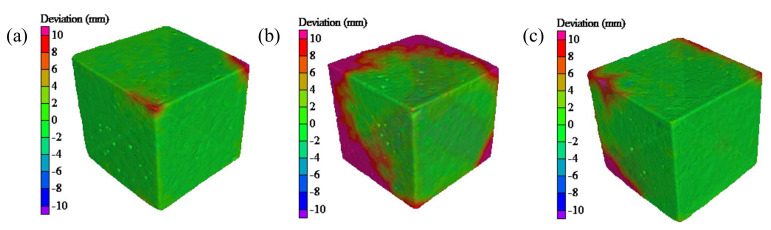
Three-dimensional deviation nephograms of concrete specimens: (**a**) Group A; (**b**) Group B; (**c**) Group C.

After 3D reconstruction, the apparent state of concrete after the salt freezing–thawing cycles and the initial apparent state were compared. Apparent deviations of concrete before and after the salt freezing–thawing cycles were discussed, in which a greater deviation indicated the higher material loss depth of concrete. The nephograms of the deviations of Groups A, B, and C after 300, 300, and 200 salt freezing–thawing cycles are shown in Figure 13. As can be seen, the red region is the material loss region of concrete, and the left scale indicates the deviation distances represented by different colors.

As shown in Figure 13, after 300 salt freezing–thawing cycles, the surface deviation of Group A was relatively small and mainly occurred at the corners. Group B showed greater deviation in a region larger than 10 mm at the corners. In Group C, deviations in some regions exceeded 10 mm after 200 freezing–thawing cycles.

**Figure 14:**
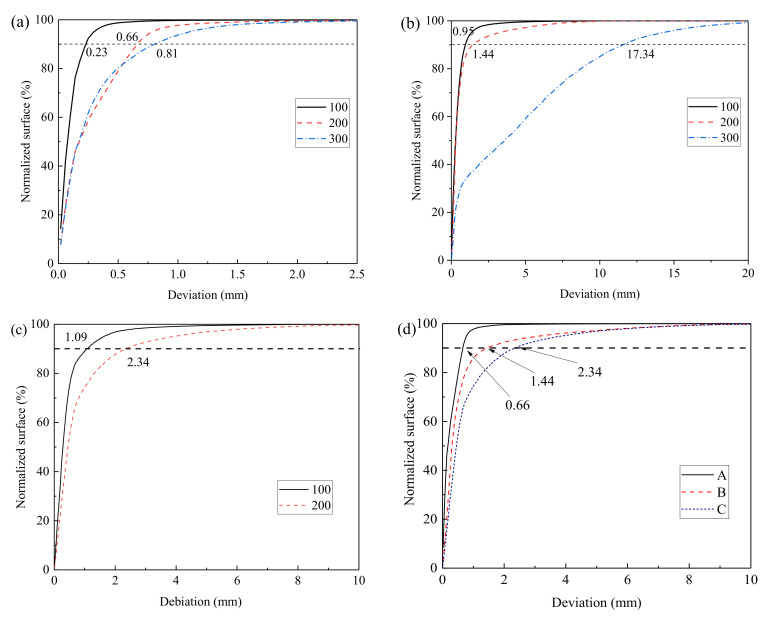
Deviation distributions of different groups: (**a**) Group A; (**b**) Group B; (**c**) Group C; (**d**) deviations of different groups after 200 cycles.

For the quantitative analysis of apparent deviations, the relation curve between concrete surface deviation and proportion of concrete surface was plotted (Figure 14). After the salt freezing–thawing cycles, surface loss of concrete often presented great discreteness. Therefore, 90% of the concrete surfaces were used as the reference in analyzing the maximum deviation, which could reflect the material loss degree of concrete to some extent.

As shown in Figure 14a–c, by using 90% surface as the reference, the maximum deviations of Group A were 0.23, 0.66, and 0.81 mm and those of Group B were 0.95, 1.44, and 17.34 mm after 100, 200, and 300 salt freezing–thawing cycles, respectively. In addition, the maximum deviations of Group C after 100 and 200 cycles were 1.09 and 2.34 mm, respectively. The test results demonstrated that with the increase in salt freezing–thawing cycles, the surface deviation of concrete increased gradually. In addition, the maximum deviations of different groups under the same number of salt freezing–thawing cycles were compared, and the results after 200 salt freezing–thawing cycles were analyzed, as shown in Figure 14d. The maximum deviations of Group A, B, and C after 200 salt freezing–thawing cycles were 0.66, 1.44, and 2.34 mm, respectively, indicating that surface deviation of concrete was positively related to the water–cement ratio. Deviation was also analyzed by material loss depth of concrete, which further disclosed the damage evolutionary rules of concrete under salt freezing–thawing cycles.

## 4. Damage Model and Damage Mechanism of Concretes

### 4.1. Construction of Damage Model

Generally, after chlorine salt erosion and freezing–thawing cycles, concrete can develop damage due to volume loss and changes in pore structure and compaction degree. The intensifying damage can significantly influence the mechanical properties of concrete. The damage variable is a state variable in materials, and its determination is the primary task of damage model construction. With respect to concrete durability, relevant scholars often used RDME as the damage variable. However, this method cannot evaluate damages from the perspective of microstructural evolution. Thus, to determine the evolutionary rules of the internal microstructure of concrete under salt freezing–thawing cycles, the compaction degree was used as the damage variable to analyze the damage evolution of concrete by combining with the CT test.

At CT scanning, concrete was divided into m × n individual elements so that m × n values of *I* could be gained. Through the reconstruction of computer images, the CT images composed of m × n attenuation coefficients could be gained. Through the linear normalization of attenuation coefficient relative to water, the compaction degree of concrete can be expressed by the CT value as follows [35]:

(1) $$H=1000\times \frac{\mu -\mu_{w}}{\mu_{w}}$$

where *H* is the CT value of concrete (Hu), *μ* is the attenuation coefficient of concrete, and *μ_w_* is the attenuation coefficient of water.

If the measured substance is a mixture, the volume percentages of the constituent materials are $V_{1}$, $V_{2}$, $V_{3}$…$V_{n}$, respectively, there is [36]:

(2) $$\mu =\sum_{i=1}^{n}v_{i}\mu_{i}$$

where $v_{i}$ is the volume fraction of the *i* material component, and $\mu_{i}$ is the attenuation coefficient of component *i* of the material.

The overall attenuation coefficient of concrete represents the average value of each component material. From the perspective of material characteristics, the composition of internal materials of concrete is divided into three states: solid, liquid, and air. Therefore, the overall attenuation coefficient is mainly composed of three parts, which can be expressed as:

(3) $$\mu =\frac{V_{s}}{V}\mu_{s}+\frac{V_{l}}{V}\mu_{l}+\frac{V_{g}}{V}\mu_{g}$$

(4) $$\left(\frac{H}{1000}+1)=\frac{V_{s}}{V}(\frac{H_{s}}{1000}+1)+\frac{V_{l}}{V}(\frac{H_{l}}{1000}+1)+\frac{V_{g}}{V}(\frac{H_{g}}{1000}+1\right)$$

(5) $$H=\frac{H_{s}V_{s}+H_{l}V_{l}+H_{g}V_{g}}{V}$$

where $\mu_{s}$, $\mu_{l}$, and $\mu_{g}$ are the attenuation coefficients of solid, liquid, and air phases in concrete, respectively; *V_s_*, *V_l_*, and *V_g_* are the volume of solid, liquid, and air phases in concrete (mm^3^); *V* is the overall volume of concrete (mm^3^); and *H_s_*, *H_l_*, and *H_g_* are CT values of solid, liquid, and air phases in concrete, respectively (Hu).

Under salt freezing–thawing cycles, CT value can only represent the compaction degree of concrete at a moment after volume loss of concrete, but it cannot reflect the degree of variation of the compaction degree. For comparison between compaction degree after salt freezing–thawing cycles and initial value, the concept of relative CT value was introduced. When the concrete was in the drying state, it was hypothesized that material loss of concrete is filled by air. Therefore, the relative CT value of concrete after a salt freezing–thawing cycle can be expressed as:

(6) $$H_{\left(n\right)}^{\prime }=\frac{H_{s(n)}V_{s(n)}+H_{g}(V_{g(n)}+V_{z(n)})}{V_{\left(0\right)}}=\frac{\left(H_{s(n)}+1000)V_{s(n)}-1000(V_{g(n)}+V_{\max}\right)}{V_{\left(0\right)}}$$

where $H_{\left(n\right)}^{\prime }$ is the relative CT value of concrete after *n* salt freezing–thawing cycles (Hu); $H_{s(n)}$ is the solid CT value in concrete after *n* salt freezing–thawing cycles (Hu); $V_{s(n)}$ is the solid volume in concrete after *n* salt freezing–thawing cycles (mm^3^); $V_{g(n)}$ is the pore volume in concrete after *n* salt freezing–thawing cycles (mm^3^); $V_{z(n)}$ is the concrete loss volume after *n* salt freezing–thawing cycles (mm^3^); $V_{\left(0\right)}$ is the initial volume of concrete (mm^3^); and $V_{\max}$ is the maximum volume of solid material during freezing-thawing cycles (mm^3^).

**Figure 15:**
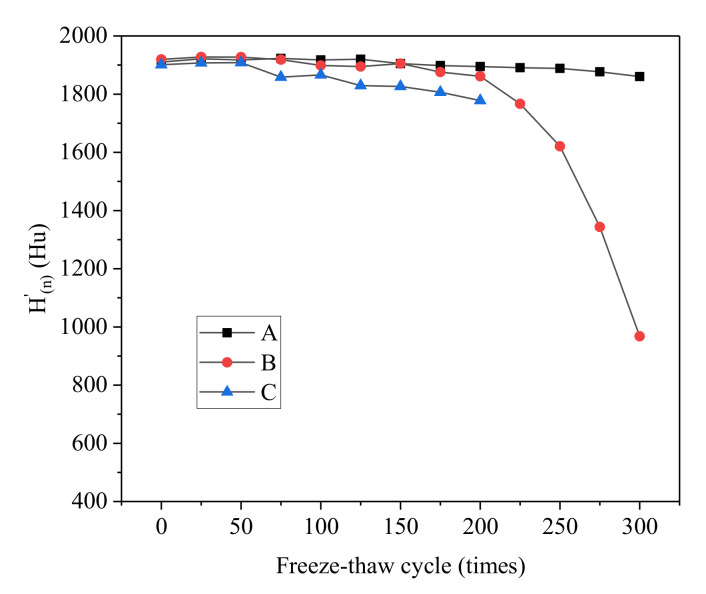
Evolution of the relative CT values.

From Equation (6), the relative CT values of concrete ($H_{\left(n\right)}^{\prime }$) after different salt freezing–thawing cycles can be calculated. The variation curves of the relative CT values of different groups with the number of salt freezing–thawing cycles are shown in Figure 15.

It can be seen from Figure 15 that the relative CT values of different groups decreased with the increase in the number of salt freezing–thawing cycles. Moreover, the reduction amplitude was positively related to the water–cement ratio. The variation trend of relative CT values was highly correlated with the damage degree of concrete specimens, thus demonstrating the macroscopic evolutionary rules of concrete during the salt freezing–thawing cycles. Hence, relative CT value was chosen as the damage variable by which to analyze the salt freezing-induced concrete damage. The ratio between relative CT values before and after salt freezing–thawing cycles was used to characterize the degree of damage (*D_n_*), expressed below.

(7) $$D_{n}=1-\frac{H_{\left(n\right)}^{\prime }}{H_{\left(0\right)}^{\prime }}$$

It has been proposed [37] that the Weibull model can accurately describe the damage process of concrete throughout freezing–thawing cycles. Hence, the two-parameter Weibull probability distribution function was used for fitting the concrete damage degree:

(8) $$D_{n}=1-\exp\left[-{\left(\frac{n}{\alpha }\right)}^{\beta }\right]$$

where α is the scale factor, β is the Weibull shape factor, and *n* is number of salt freezing–thawing cycles.

**Figure 16:**
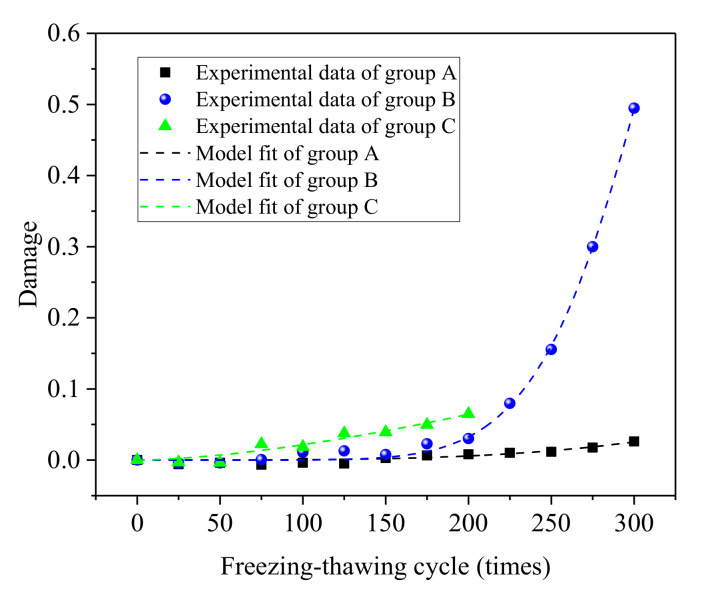
Comparison between damage model and test results.

Based on the fitting of test data, the Weibull damage evolutionary equations of groups A, B, and C after different numbers of salt freezing–thawing cycles could be gained using Equations (9)–(11). The comparison between the calculated results of the damage model and test value is shown in Figure 16.

(9) $$D_{n}=1-\exp\left[-{\left(\frac{n}{810.05}\right)}^{3.68}\right]\;(R^{2}=0.861)$$

(10) $$D_{n}=1-\exp\left[-{\left(\frac{n}{315.88}\right)}^{7.45}\right]\;(R^{2}=0.998)$$

(11) $$D_{n}=1-\exp\left[-{\left(\frac{n}{1081.71}\right)}^{1.61}\right]\;(R^{2}=0.940)$$

As shown in Figure 16, the concrete damage increased gradually with the increase in the number of salt freezing–thawing cycles. Given the same salt freezing–thawing cycles, concrete damage was positively related to the water–cement ratio. The damage model that was constructed according to the Weibull probability distribution agreed well with the test results. The correlation coefficient between the model curves of damage equation and the test results of different groups was higher than 0.86, indicating that the constructed model could well describe the damage evolution of concrete during the salt freezing–thawing cycles.

### 4.2. Damage Mechanism Analysis

Under the coupling effect of chlorine salt erosion and freezing–thawing cycles, concrete bears chemical erosion and physical erosion simultaneously. Chemical erosion is mainly caused by chemical reactions between chlorine salt and hydration products found in the concrete [38]. In hydration products of concrete, it keeps losing Ca(OH)_2_ in the chlorine salt environment, as its solubility increases, thereby increasing porosity in concrete and inducing concrete damages. Furthermore, Cl^−^ can react with C_3_A in concrete to produce C_3_A·CaCl_2_·10H_2_O. As the volume of reaction products is higher than the original structure, a swelling stress is produced in concrete, thus changing the microstructure and finally causing concrete damages. The major chemical reactions are shown below.

(12) $$2NaCl+Ca{\left(OH\right)}_{2}=CaCl_{2}+2Na^{+}+2OH^{-}$$

(13) $$C_{3}A+CaCl_{2}+10H_{2}O=C_{3}A\cdot CaCl_{2}\cdot 10H_{2}O$$

Physical erosion mainly involves frost heaving and permeability. In freezing–thawing cycles, volume swells upon icing of water in the concrete pores. When swelling stress exceeds the compressive strength of concrete, cracks initiate in the concrete. With the increase in the number of freezing–thawing cycles, cracks propagate gradually and connect mutually, after which a failure surface is formed, thus causing falling of mortar and aggregate [39]. In a chlorine environment, there’s a concentration difference between the internal and external pores in the concrete, resulting in increased solution absorption mass by pores in the concretes. Hence, more pores produce such frost heaving damages, which, in turn, accelerate the concrete damages. In addition, a larger concentration difference is formed between the internal structure of concrete and the external environment after icing of the solution. The solution penetrates from the outer to the inner part continuously, which produces an osmotic pressure to intensify concrete failure.

In the salt freezing–thawing cycle test, concrete with different water–cement ratios showed different frost resistance levels. The frost resistance has been shown to be lower when the water–cement ratio is higher. This could be interpreted based on the permeability and energy of concrete [40]. On the one hand, the internal materials of concrete become looser when the water–cement ratio is higher; thus, permeability is stronger, and there are more internal connected pores. When concrete is immersed in a solution, it would be easier for the solution to penetrate into concrete, causing greater damage during the freezing–thawing cycles. On the other hand, because less energy is required for concrete cracking during the freezing–thawing cycles, frost resistance is lower when the damage is more serious given the same salt freezing–thawing cycles.

## 5. Conclusions

The macro–meso damage evolutionary rules of concrete under the coupling effect of chlorine salt erosion and freezing–thawing cycles were analyzed comprehensively in this work. The following conclusions are drawn:

(1) Concrete surface experienced three stages of microcrack initiation, mortar peeling, and aggregate falling during the salt freezing-thawing cycles. The mass loss rate decreases in the beginning and then increases in the process of chlorine salt erosion and freezing–thawing, and the smaller the concrete size, the greater the mass loss rate.
(2) The relative dynamic modulus of elasticity decreases gradually, slowly at the initial stage and then at a faster rate, and the compressive strength loss rate increases gradually. Comprehensive analysis showed that the frost resistance of concrete is negatively related to the water-cement ratio when the freezing–thawing cycle is fixed.
(3) The evolution of concrete micro-structure during the salt freezing–thawing cycles can be quantified by CT technology. The pore quantity, porosity, and volume loss rate of concrete increase in a fluctuating manner, whereas the relative CT value decreases. The peeling depth of the concrete surface increased gradually. The higher the water-cement ratio is, the greater the change of micro-structure will be.
(4) The damage model was established with relative CT value as the damage variable, and the model could better reflect the freezing–thawing damage degree of concrete with different water cement ratios, and the damage evolution process could be well described by the Weibull function.

## Figures and Tables

**Table 1:** Physical and mechanical properties of cement.

Initial/Final Setting Time (min)	Density (g/cm^3^)	Specific Surface Area (m^2^/kg)	Standard Consistency Water Consumption (%)	Flexural Strength (MPa)		Compressive Strength (MPa)	
				3d	28d	3d	28d
65/260	3.05	358	28.8	5.6	8.6	29.5	51.0

**Table 2:** Mix proportions and mechanical properties.

Samples	Water–Cement Ratio	Cement (kg/m^3^)	Sand (kg/m^3^)	Gravel (kg/m^3^)	Water (kg/m^3^)	7d Compressive Strength (MPa)	28d Compressive Strength (MPa)
A	0.35	571	600	1160	200	29.8	50.5
B	0.45	444	620	1200	200	25.8	39.2
C	0.55	364	670	1240	200	18.8	32.6

## Data Availability

Not applicable.
